# The profile of head injuries and traumatic brain injury deaths in Kashmir

**DOI:** 10.1186/1752-2897-2-5

**Published:** 2008-06-21

**Authors:** GH Yattoo, Amin Tabish

**Affiliations:** 1 Department of Hospital Administration, Sher-i-Kashmir Institute of Medical Sciences, Srinagar, India; 2 Accident & Emergency Department, Sher-i-Kashmir institute of Medical Sciences, Srinagar, India

## Abstract

This study was conducted on patients of head injury admitted through Accident & Emergency Department of Sher-i-Kashmir Institute of Medical Sciences during the year 2004 to determine the number of head injury patients, nature of head injuries, condition at presentation, treatment given in hospital and the outcome of intervention. Traumatic brain injury (TBI) deaths were also studied retrospectively for a period of eight years (1996 to 2003).

The traumatic brain injury deaths showed a steady increase in number from year 1996 to 2003 except for 1999 that showed decline in TBI deaths. TBI deaths were highest in age group of 21–30 years (18.8%), followed by 11–20 years age group (17.8%) and 31–40 years (14.3%). The TBI death was more common in males. Maximum number of traumatic brain injury deaths was from rural areas as compared to urban areas.

To minimize the morbidity and mortality resulting from head injury there is a need for better maintenance of roads, improvement of road visibility and lighting, proper mechanical maintenance of automobile and other vehicles, rigid enforcement of traffic rules, compulsory wearing of crash helmets by motor cyclist and scooterists and shoulder belt in cars and imparting compulsory road safety education to school children from primary education level. Moreover, appropriate medical care facilities (including trauma centres) need to be established at district level, sub-divisional and block levels to provide prompt and quality care to head injury patients

## Introduction

Head injury is considered as a major health problem that is a frequent cause of death and disability and makes considerable demands on health services. In developing countries accident rates in general and traumatic brain injury in particular are increasing as traffic increases besides other factors like industrialization, falls and ballistic trauma. Head injuries account for one quarter to one third of all accidental deaths, and for two thirds of trauma deaths in hospitals [1].

Traumatic brain Injury is a leading cause of mortality in patients younger than 45 years accounting for more than a third of all injury related deaths in United States. Each year 52,000 people die and another 80,000 suffer morbidity and traumatic brain injury. Although more severe injuries are associated with poorer outcomes, the moderately injured patients also are at risk [2].

Road traffic injury is an increasing health problem globally and especially in South-East Asia [3].

Trauma presents with variety of injuries and problems that demand rapid evaluation, discussion, improvisation and intervention to save life and prevent permanent disability. Gunshot and blast injuries are common cause or brain injury during war time with increased incidence during peace also. Civilian are the major targets in recent war situations and account for more than 80% of those wounded and killed [4].

Closed head injury is the result of variety of mechanisms including motor vehicle and motor cycle accidents, falls from heights, assaults and pedestrians being struck by motor vehicles. Penetrating injury is most often due to gunshots but sometimes other types of blunt objects can violate the skull. Most commonly, traumatic brain injury occurs in the presence of additional injuries to other major organ systems but it can also occur in isolation [2].

The management of head injury has been revolutionized by round the clock monitoring, carotid angiography, isotope brain scan, computerized axial tomography and monitoring of intracranial pressure. Intermittent positive pressure ventilation, dehydration therapy and better techniques of operations have made a tremendous difference in the ultimate outcome of severe brain trauma. Recently introduced multimodality evoked potential technique has further improved this management [5].

The violence is endemic in nature with periods of exacerbation and remissions. It is a dynamic process. War related violence is currently acknowledged as one of the most important public health problems particularly when it is used on mass scale to specifically destabilize the social and cultural infrastructure of community. In Kashmir there has been increase in incidence of ballistic trauma secondary to bullets, blasts and stabs since 1990 subsequent to civil disturbance in the state [4].

Brain injury is the most common cause of death in trauma victims accounting for about half of deaths at the accident site. The injuries are generally blunt and motor vehicle accidents are most frequent. Of particular significance are motorcycle accidents involving passengers without helmet, which produce severe injuries. As many as two thirds of all motor vehicle accident victims sustain some head injury. Complications from closed head injuries are the single largest cause of morbidity and mortality in patients who reach the hospital alive. Of patients who require long term rehabilitation, head trauma is usually the primary injury. This data is generally applicable to children as well. Although the mechanisms vary, head injuries are the major cause of morbidity and mortality in childhood trauma victims, accounting for an annual mortality rate of 1 per 1000 in this age group [6].

Today we have a better understanding of the mechanisms of both primary damage caused by the critical insult and the destructive process which are triggered by it. The monitoring technology required to detect adverse secondary events has evolved considerably in the past few years. Significant reduction in mortality and morbidity associated with severe head injury has been achieved with aggressive management protocols that emphasize maintenance of blood pressure, prompt evacuation of mass lesion and control of intracranial pressure. Current efforts in the field are directed at the development and clinical testing of new drugs and physiological intervention. It is anticipated that further improvements in outcome will be derived from the cumulative benefits of several such interventions [7].

Because a head injury occurs every 15 seconds and a patient dies from head injury every 12 minutes, a day does not pass that an emergency physician is not confronted with a head injured patient [8].

Studies concerning the frequency and occurrence of head injuries and their importance in causing death and disability solidify the impression that such injuries are important problems in emergency medicine.

Estimates of mortality following head injury vary from 10–36 per 100,000 population, accounting for up to 70,000 deaths per year in the United States. Head injuries are responsible for 2% of all deaths and 26% of injury deaths in this country. Furthermore, approximately half of the nearly 4 million days of hospitalization per year from motor vehicle trauma are for head injuries [8].

Severe head injury is associated with high mortality and morbidity [8]. Destruction of the brain is the fundamental medical and legal standard for human death. Permanent cessation of heartbeat and breathing produces death because, without resuscitative, efforts, destruction of the whole brain occurs. The term "brain death" refers to a temporary conditions where the whole brain has been destroyed but heartbeat and organ and tissue metabolism are maintained via technologic support of cardio-respiratory functions [9].

Brain death determination is usually not feasible in the emergency department (ED). It takes several hours and usually longer to stabilize the patient medically, accomplish the necessary tests, and ascertain the irreversibility of the condition. For patients with profound brain damage, however, particularly in setting of massive brain trauma, the clinician may deem the patient "unsalvageable" [9].

Unsalvageability usually refers to brain damage so profound that one of three outcomes is inevitable (a) the patient will be declared brain dead within 24 hours when formal brain-death testing is carried out, (b). the patient is not initially brain dead but will become brain-dead during the hospital admission or (c) the patient will not be declared brain-dead but will have suffered permanent brain damage so profound that, in the opinion of the clinician, he would be better off had he died initially [9].

Any therapeutic inadequacies may result in further increase in morbidity and mortality. In spite of best management, 15–20% of head injuries prove fatal. The majority of patients require conservative management and only 10–20% of patients need surgical intervention [5].

Any therapeutic inadequacies may result in further increase in morbidity and mortality. Hence management of head injury is also a responsibility, requiring sound judgment and awareness of a possibility of injury to other systems which may complicate the problems. In spite of best management, 15–20% of head injuries prove fatal. The majority of patients require conservative management and only 10–20% of patients need surgical intervention [5].

The importance of protecting the head from injury is gaining wider recognition. Firm preventive efforts will clearly be effective in reducing the incidence of serious head injuries and their complication. Compulsory wearing of protective devices e.g. helmets and automatic shoulder belts, in cars etc have reduced the incidence and degree of serious Research and epidemiological data are required to initiate appropriate preventive measures and to plan necessary services.

### Objective

The objective is to study nature of head injury patients and condition at presentation, yearly trends of traumatic brain injury deaths (1996–2003) and risk of brain injury deaths with respect to demographic variables

## Methods

This study was carried out at the Emergency Department of SKIMS, to determine the profile of head injuries and traumatic brain injury deaths of traumatic head injury.

The study was conducted into two phases. *In prospective study*, patients of head injury reporting to Accident & Emergency Department of SKIMS for a period of one year (January 2004 to December 2004) were selected by random sampling method taking sample size of 14%, i.e. one day was selected for study in a week which amounted to 52 days in year. All the patients of head injury who reported to Accident & Emergency Department were followed during their stay in the hospital from admission to discharge or death. The information regarding the patients of head injury who reported to Accident & Emergency Department was obtained from the Medical Records Department. *In retrospective study*, information from Medical Record Department was obtained regarding traumatic brain injury deaths for a period of eight years (January 1996 to December 2003). Yearly trends of traumatic brain injury deaths were noted. Risk of traumatic brain injury deaths with respect to demographic variables was also observed.

Information was obtained regarding nature of head Injury (scalp, skull, intracranial); mode of injury (fall from height, road traffic accident, fire arm injury, assault, blast injury); condition at presentation [Glasgow Coma Scale (GCS)]; treatment received (investigations) and outcome of treatment.

*The Prospective study *included 3861 patients with head injury reporting to the Accident and Emergency Department of SKIMS, from January to December 2004, to determine the condition of patients at the time of presentation based on GCS, morbidity and mortality, population at greater risk and leading cause of traumatic brain injury (TBI). Random sampling method was used and 547 patients selected for the study. *Retrospective study *included all cases of brain injury deaths during 1996 to 2003 (8 years) at SKIMS. The total number of traumatic brain injury deaths during this period was 1298 out of a total of 18093 head injury patients admitted.

The data was collected regarding age, gender, geographical distribution, occupation, nature of injury, mode of injury, condition at presentation, management and outcome of intervention.

## Results

The total number of patients of head injury admitted in Accident and Emergency Department for one year was 3861. The highest number of patients was admitted during the month of July 2004 and lowest during January, 2004. The total number of patients admitted in Accident and Emergency for 2004 is shown in Table 1.

**Table 1:** Total month-wise admissions of head injury patients in the year 2004 (n = 3861)

**Month**	**Total No. of patients (n)**	**Percentage**
January	161	4.1
February	268	6.9
March	341	8.8
April	315	8.1
May	397	10.8
June	354	9.1
July	457	11.9
August	326	8.4
September	353	9.1
October	271	7.0
November	300	7.7
December	318	8.1

**Total (12 months)**	**3861**	**100%**

The age of patients varied from 6 months to 80 years. The majority belonged to age group 0–10 years followed by age group 21–30 years. The age distribution is given in Table 2.

**Table 2:** Admissions according to age of patients incorporated in the study (n = 547)

**Age in years**	**Total No. of patients**	**Percentage**
0–10	139	25.5
11–20	85	15.5
21–30	116	21.2
31–40	100	18.2
41–50	39	7.1
51–60	43	7.8
61–70	16	3.0
71–80	9	1.7

**Total**	**547**	**100%**

Highest incidence of head injury has been reported in the age group of 2–10 years by most of authors. [5] The age of patients varied from 6 months to 80 years. Majority of patients were found to be in the first-decade of life (25.5%). The second highest age group was in third decade (21.2%) followed by fourth decade (18.2%) and second decade (15.5%) respectively. These being the active group of society who spend most of their time out of their houses for education and to earn the livelihood are more prone to accidents [3].

The overall male: female ratio being 3:1. Most of patients of head injury are from rural areas. The maximum number of patients came from Srinagar city followed by Baramulla town and other districts. Majority of patients admitted were labourers followed by students. The distribution as per occupation is shown in Table 3.

**Table 3:** Occupation-wise distribution

Occupation	No. of patients	Percentage
Students	129	23.6
Labourers	143	26.1
House wives	68	12.4
Businessmen	35	6.4
Government employee	29	5.3
Farmer	61	11.2
Others	82	15.0

Total	547	100%

Scalp lacerations constituted the bulk of such patients. The nature of injury distribution is shown in Table 4.

**Table 4:** Nature of Head Injury

**Nature of Injury**	**No. of patients**	**Percentage**
Scalp Laceration	221	40.4
Commuted fracture of skull	32	5.9
Depressed fracture of skull	16	3.0
SDH	23	4.2
EDH	18	3.2
Brain Contusions	48	8.8
SAH	2	0.3
ICH	2	0.3
Brain edema	7	1.3
No external/internal injury	103	18.9
Multiple aerocele	5	0.9
Cephalohaematoma	34	6.2
Both	36	6.6
**Associated Injuries**	**46**	
Chest injury	09	
Abdominal injury	10	
Skeletal Injury	27	

Road traffic accident was the main cause of head injury followed by fall from height. The distribution of patients according to cause of injury is shown in Table 5.

**Table 5:** Mode of Injury

Mode of Injury	No. of patients	Percentage
Fall from height	176	32.2
Road Traffic accident (RTA)	243	44.4
Assault	103	18.8
Blast Injury	21	3.8
Fire Arm Injury	4	0.8

Total	547	100%

At the time of admission 80.2% of patients were conscious having GCS of 15. The GCS distribution is shown in Table 6.

**Table 6:** Glasgow coma scale score of 547 head injury patients

Glasgow Coma Scale score	No. of patients	Percentage
15	439	80.2
13–14	54	9.9
8–12	29	5.3
<8	25	4.6

Total	547	100

X-ray was done in all patients and CT scan head (plain) was done in 547 patients. The results were normal in 499 and 32 had linear fracture and 16 showed depressed fracture (Table 7).

**Table 7:** Results of CT scan of head (plain) in 461 patients

**Nature of Injury**	**No.**
Acute SDH	23
EDH	18
Contusion	48
Brain edema	7
Multiple aeorocele	5
Linear fructure	32
Depressed fracture	16
Both (Skull # and brain injury)	36
SAH	2
ICH	2
Normal study	272

Total	461 (84.2%)

91.54% (495) patients were treated conservatively and 9.5% (52) needed surgical intervention. Majority of patients admitted improved which is shown in Table 8.

**Table 8:** Outcome of treatment

**Glasgow coma scale score**	**No. of patients**	**Improved**	**Expired**
15	439	439	-
13–14	54	51	3
8–12	29	22	7
<8	25	-	25

Total	547	512	35

*Retrospective study *included all cases of brain injury deaths during 1996 to 2003 (8 years). The total number of traumatic brain injury deaths during this period was 1298 out of a total of 18093 head injury patients admitted.

The highest number of deaths was seen in 1998 with lowest number of deaths in 2000 and 2001. The yearly trends of Traumatic Brain Injury (TBI) death percentage is shown in Table 9.

**Table 9:** Yearly trends of TBI deaths

Year	Total No. of patients	No. of deaths	
		
		No.	%
1996	1629	130	7.9
1997	1810	146	8.0
1998	1900	159	8.3
1999	1872	132	7.0
2000	2241	136	6.0
2001	2710	160	6.0
2002	2826	205	7.2
2003	3105	230	7.4

Total (1996–2003)	18093	1298	7.17%

CT scan findings are shown in Table 10. Majority of the patients presented with a fracture and epidural bleeding or a contusion with subarachnoidal bleeding and brain oedema. Isolated injuries were seen occasionally.

**Table 10:** CT scan finding of 1298 patients who died from 1996 to 2003

**CT scan findings**	**No. of patients**
EDH	103
SDH	149
Contusion	845
Brain edema	40
SAH	35
ICH	47
# Base of skull	28
Diffuse axonal injury	51

EDH (extradural hematoma), SDH (subdural hematoma), SAH (sub-arachnoid hemorrhage), ICH (intracranial hemorrhage)

The number of TBI deaths was highest in the age group of 31–40 and lowest in age group of 71–80 (Table 11).

**Table 11:** Age and TBI deaths

**Age (years)**	**Year-wise deaths**								**Total deaths**	**Percentage**
			
	1996	1997	1998	1999	2000	2001	2002	2003		
0–10	16	11	15	18	20	20	28	24	152	11.7
11–20	9	21	27	12	13	19	18	16	135	10.4
21–30	29	37	30	19	23	26	29	37	230	17.8
31–40	26	21	28	20	27	34	37	49	243	18.8
41–50	30	21	20	24	15	27	38	40	215	16.5
51–60	12	23	26	21	25	17	30	32	186	14.3
61–70	6	9	9	12	10	12	18	26	102	7.8
71–80	2	3	4	6	3	5	6	6	35	2.7

The TBI deaths are more common in males as compared to females (Table 12).

**Table 12:** Gender and TBI deaths

	**Sex**	**Year**							
		
		1996	1997	1998	1999	2000	2001	2002	2003
Total No. of TBI patients	Male	1228	1390	1450	1442	1720	2100	2126	2376
Total TBI deaths	Male	111	124	133	111	106	134	163	187
Percentage		9	8.9	8.5	9.1	6.1	6.4	7.6	7.9
Total No. of TBI patients	Female	401	420	450	430	521	610	700	729
Total TBI deaths	Female	19	22	26	21	30	26	42	43
Percentage		4.7	5.2	5.7	4.9	5.7	4.2	6	5.8

TBI deaths are more common in rural as compared to urban areas (Table 13).

**Table 13:** Geographical Distribution of TBI deaths

	**Area**	**Year**							
		
		1996	1997	1998	1999	2000	2001	2002	2003
Total No. of TBI patients	Rural	1303	1448	1520	1498	1793	2168	2260	2484
Total TBI deaths	Rural	100	109	124	96	105	130	162	182
Percentage		7.6	7.5	8.5	6.4	5.8	5.9	7.1	7.3
Total No. of TBI patients	Urban	326	362	380	374	448	542	566	621
Total TBI deaths	Urban	30	37	35	36	31	30	43	48
Percentage		9.2	10.0	9.2	9.6	6.9	5.5	7.5	7.7

Majority of the patient who died due to head trauma belonged to Districts of Anantnag, Ladakh and Baramulla.

## Discussion

Kashmir has a population of population of Kashmir Province (the size of the population at risk that may be referred to SKIMS hospital) is 57, 13,509 (2001 census) out of which 27, 07,837 are females. Kashmir province has 11 hospitals associated to Medical Colleges, 8 District Hospitals, 47 Community Health Centres, 661 Primary Health Centres/Allopathic Dispensaries, 1105 Sub Centres, 184 Indian System of Medicine (Unani) dispensaries and, 21 other healthcare institutions. Health care is supported by the private sector hospitals, nursing homes, poly clinics, GP clinics, faith healers, quacks, etc. Facilities for treating head trauma are available only at SKIMS.

The annual incidence rate of head injuries (n/100,000) is 55/100000 (approximate).

The annual overall numbers of admissions and trauma admissions to the Emergency Department at SKIMS hospital is 18327 and 3861 (during 2004) respectively.

Several studies in other parts of the world indicate a steady rising incidence of head injuries (34). One in 12 deaths in United States results from injury, with over 147,505 injury related death in 1994. The exact proportion of those deaths that result from brain injury is not known but if mortality estimates from Olmsted County, Minnesota, San Diego County, California and data from National Center for Health Statistics are averaged, the annual fatality rate from brain injuries is about 25/100000/year or roughly 46% of overall injury death rate of 57/100000/year for 1994. A somewhat similar figure has been reported from Australia, but a recent report by Sosin et al estimate the proportion of head injury related deaths in the United States to be 28% of all injury deaths [10].

Highest incidence of head injury has been reported in the age group of 2–10 years by most of authors [5]. Age specific rates for deaths and admission from head injury in U.K. across all ages shows the peak incidence in the 15–30 years age range but for attenders at Accident and Emergency Department this peak is younger than 10 years [1]. Frank Kennedy et al in their series of 192 patients reported that 90% were 0–14 years old [11].

In our study the age of patients varied from 6 months to 80 years. Majority of patients were found to be in the first-decade of life (25.5%). Children play in the streets, have less supervision of the parents and above all there is lack of safety measures in place where they play. Males predominate in the present study also. The male: female ratio being 3:1. Most of the USA reports show an incidence ratio of 2.0 or more for males compared to females [10]. Adam et al (1996), in their series of 672 patients showed 533 was male and 139 female patients [12]. Our observation corresponds with the observations made by other authors. The reason is that males move out of their homes more frequently and are more actively working than females.

Gabella B, et al used a State Surveillance System to identify cases of traumatic brain injury for the year 1991 and 1992. The study showed higher rates of severe traumatic brain injury in rural as compared to urban areas [13]. Our study also shows higher rates of traumatic brain injuries in rural (71.1%) than urban (28.9%) areas. The reasons are: less awareness, injury due to fall from height being common and of course ballistic trauma. The poor outcome is attributed to ignorance, poor infrastructure including mode of transportation, lack of proper health care facilities, distance to reach to the appropriate hospital, poverty, etc. SKIMS is the only healthcare facility in Kashmir (referral centre) where head trauma is being treated. The outcome largely depends on prompt and quality care to such patients which is deficient in rural areas.

In the present study, highest number of patients was having scalp lacerations (40.4%), contusion (8.8%), EDH (3.2%), SDH (4.2%) and depressed fracture (3%). Early detection of Extradural Hematoma (EDH), Subdural hematoma (SDH) and depressed fracture will improve outcome of these patients by early surgical intervention [14].

Eight of ten in the USA incidence reports showed transport related events as single largest cause of new cases of brain injury. Falls routinely are the second leading cause in most incidence studies [10]. Shin Tseng Lee et al in their series of 10,000 patients from 1977–1987 admitted to Chand Gung Memorial Hospital in Taiwan observed 56.3% were motorcycle accidents [15]. In the present study, the mode of injury showed Road Traffic Accidents responsible for 44.4% followed by falls 32.2% (Table 14).

**Table 14:** Percentage distribution of brain injuries by external causes

Place (year)	Mode of injury				
	
	Trans-port	Fall	Firearm or assault	Sports	Others
New Delhi (1969)	35	49	9	-	7
Washington (1974)	14	33	37	-	16
India (1974)	21	60	10	-	9
Taiwan (1990)	90	5	-	-	5
Pakistan	50	16	22	-	3
Kashmir (this study: 2004)	44.4	32.2	19.6	-	3.9

In 1978 it was suggested that the Glasgow Coma Scale (GCS) be used to assess the seriousness of head injury, that the criteria used by the International Data Bank. (a total GCS score of 8 or less for 6 hours) be used to set the boundaries of patient study groups, and that the Glasgow outcome scale be used as the initial end point at a specified time from injury for measuring morbidity and mortality [16].

In the present study 80.2% patients were having normal GCS and 4.6% were having GCS < 8 (severe grade).

Out of 547 patients studied, X-ray skull was done in all patients (100%) and CT scan head was done in 84.2% of patients. For any hospital with neurosurgeons available, CT scanning is an important, which dramatically improves management and outcome of head injury patients.

Knut Wesler, et al, in their study observed by introducing a new diagnostic tool, a CT scanner in a Norwegian County hospital resulted in a marked redistribution of the handling of acute/severe head injuries [17].

Out of 547 patients in this study, 93.6% improved and 6.4% expired. The outcome of head injury can be improved by removal of space occupying hematomas at the earliest time possible and by early vigorous management of intracranial hypertension [16].

Sosin DM, et al used Multiple Cause-Deaths Public Use Data Tapes for 1979–1986 for National Center for Health Statistics to identify major causes of head injury associated deaths and those persons who are at a greater risk in United States [18].

There were 3,15,328 deaths associated with head injury during the period under review representing 2% of all deaths and 26% of fatal injuries.

The annual rate of head injury associated death was 16.9/100,000. Motor vehicle causes 57% of these deaths, firearm and falls were next most frequent cause. Mortality peaked at the age of 15–24 years [18].

Julie L, et al in their study published in MMWR (December 06, 2002) reveal that during 1989–1998 an annual average of 53, 288 deaths among the U.S. residents were associated with TBI for a rate of 20.61/100,000 population. During 1989–1998 TBI related deaths declined in all age groups. TBI related deaths were 3 times higher for males as compared to females [19]. Rural residents appear to have higher rates of severe traumatic brain injury deaths [20].

In the present study, the TBI deaths showed a steady increase from 1996 to 2003 except during the year 1999 which showed a decline in number of TBI deaths as compared to 1998. This increase is mostly due to number of vehicles, mixed traffic conditions. Enforcement agencies do not work in coordination and are willing to compromise, tree climbing to earn livelihood, not using helmet, not using seat belts while driving, absence of driver training and civil disturbance in Kashmir. In this study, maximum number of deaths was in third decade 18.8% followed by second decade 17.8%. Males showed increased TBI death percentage as compared to females. Maximum number of TBI deaths was from rural areas. The average TBI deaths in our study are 7.17%.

## Conclusion

This study reveals that the head injury is one of the major health problems in Kashmir. The age of head injury patients varied from 6 months to 80 years and the maximum number of patients (25.5%) was found in the first decade of life. There was preponderance of head injury in males. Maximum number of patients admitted was from Srinagar 36.2%. Majority of patients were labourers 26.1% followed by students 23.6% and house wives 12.4%. The mode of injury in majority of patients was Road Traffic Accident 44.4% followed by fall from height 32.2%, assault 18.8%, blast injury 3.8% and fire arm injury 0.8%. The nature of injury in majority of patients was scalp lacerations 40.40%, commuted fracture 5.9%, contusion 8.8%, SDH 4.2%, EDH 3.2%, depressed fracture 3%, brain edema 1.3%, SAH 0.3% and ICH 0.3%. Majority of patients were having normal Glasgow Coma Scale. Conservative treatment was given in majority of patients 90.5% followed by surgical treatment (9.5%). Majority of patients improved (93.6%) and (6.4%) expired after treatment.

Appropriate medical care facilities (including trauma centres) need to be established at district level, sub-divisional/tehsil level and block levels to provide prompt and quality care to head injury patients. The facilities at referral centre (SKIMS) for the treatment of head injury patients need to be upgraded to cater to ever-increasing number of such patients.

## Competing interests

The authors declare that they have no competing interests.

## Authors' contributions

YGH participated in the design of the study, collected data and performed the statistical analysis.

AT conceived of the study, and participated in its design and coordination.

All authors read and approved the final manuscript.
